# The coronavirus pandemic impact on involuntary hospitalization

**DOI:** 10.1192/j.eurpsy.2021.299

**Published:** 2021-08-13

**Authors:** C. Oliveira, F. Caldas, M. Gonçalves, J. Freitas

**Affiliations:** Internamento C, Hospital de Magalhães Lemos, Porto, Portugal

**Keywords:** COVID-19, compulsory admission, mental health impact, involuntary hospitalizations

## Abstract

**Introduction:**

Compulsory admission is the last line of intervention in individuals who suffer from severe mental disorders, based on the principles of therapeutic need and social protection. In Portugal, the law configures this measure as a hospitalization by court order. The SARS-CoV-2 coronavirus is a global public health emergency, with multifaceted consequences for people’s lives and health. Several studies are showing a great impact of the pandemic on the overall mental health.

**Objectives:**

The aim is to assess the impact of the pandemic on the number of involuntary hospitalizations, their socio-demographic and clinical characteristics, and study the differences between 2019 and 2020.

**Methods:**

Consultation of all patient’s clinical files that were involuntarily admitted in Hospital Magalhães Lemos during 2019 and 2020. Statistical analysis of data.

**Results:**

The authors claim to investigate the impact of the pandemic on involuntary hospitalizations, the factors of admission and decompensation and other clinical characteristics, by comparing the involuntary hospitalizations during 2019 and 2020. The authors believe that the number of compulsory admissions increased significantly with the pandemic. They also believe that factors such as increased or relapsed consumption of alcohol and drugs, suicide attempts, missed appointments and long-term injectable medication are at the root of this increase in 2020.

**Conclusions:**

This study helps to analyze the impact of the new coronavirus on compulsory hospitalizations and allows to understand the main factors that aggravate the underlying pathologies. Thus, understanding the targets of greater attention from psychiatrists to avoid the decompensation of patients in times of pandemic in which we currently live.

**Disclosure:**

No significant relationships.

